# *De novo* assembly and characterization of the transcriptome of the toxic dinoflagellate *Karenia brevis*

**DOI:** 10.1186/1471-2164-15-888

**Published:** 2014-10-11

**Authors:** Darcie E Ryan, Alan E Pepper, Lisa Campbell

**Affiliations:** Department of Oceanography, Texas A & M University, College Station, TX 77843 USA; Department of Biology, Texas A & M University, College Station, TX 77843 USA

**Keywords:** *De novo* transcriptome assembly, Harmful algal bloom, Osmoacclimation, Cation channel, Comparative transcriptomics

## Abstract

**Background:**

*Karenia brevis* is a harmful algal species that blooms in the Gulf of Mexico and produces brevetoxins that cause neurotoxic shellfish poisoning. Elevated brevetoxin levels in *K. brevis* cells have been measured during laboratory hypo-osmotic stress treatments. To investigate mechanisms underlying *K. brevis* osmoacclimation and osmoregulation and establish a valuable resource for gene discovery, we assembled reference transcriptomes for three clones: Wilson-CCFWC268, SP3, and SP1 (a low-toxin producing variant). *K. brevis* transcriptomes were annotated with gene ontology terms and searched for putative transmembrane proteins that may elucidate cellular responses to hypo-osmotic stress. An analysis of single nucleotide polymorphisms among clones was used to characterize genetic divergence.

**Results:**

*K. brevis* reference transcriptomes were assembled with 58.5 (Wilson), 78.0 (SP1), and 51.4 million (SP3) paired reads. Transcriptomes contained 86,580 (Wilson), 93,668 (SP1), and 84,309 (SP3) predicted transcripts. Approximately 40% of the transcripts were homologous to proteins in the BLAST nr database with an E value ≤ 1.00E-6. Greater than 80% of the highly conserved CEGMA core eukaryotic genes were identified in each transcriptome, which supports assembly completeness. Seven putative voltage-gated Na^+^ or Ca^2+^ channels, two aquaporin-like proteins, and twelve putative VATPase subunits were discovered in all clones using multiple bioinformatics approaches. Furthermore, 45% (Wilson) and 43% (SP1 and SP3) of the *K. brevis* putative peptides > 100 amino acids long produced significant hits to a sequence in the NCBI nr protein database. Of these, 77% (Wilson and SP1) and 73% (SP3) were successfully assigned gene ontology functional terms. The predicted single nucleotide polymorphism (SNP) frequencies between clones were 0.0028 (Wilson to SP1), 0.0030 (Wilson to SP3), and 0.0028 (SP1 to SP3).

**Conclusions:**

The *K. brevis* transcriptomes assembled here provide a foundational resource for gene discovery and future RNA-seq experiments. The identification of ion channels, VATPases, and aquaporins in all three transcriptomes indicates that *K. brevis* regulates cellular ion and water concentrations via transmembrane proteins. Additionally, > 40,000 unannotated loci may include potentially novel *K. brevis* genes. Ultimately, the SNPs identified among the three ecologically diverse clones with different toxin profiles may help to elucidate variations in *K. brevis* brevetoxin production.

**Electronic supplementary material:**

The online version of this article (doi:10.1186/1471-2164-15-888) contains supplementary material, which is available to authorized users.

## Background

The dinoflagellate *Karenia brevis* blooms almost annually in the Gulf of Mexico and is the region’s major harmful algal species [1]. *K. brevis* produces two ladder-frame polyether brevetoxin compounds, PbTx-1 and PbTx-2, which bind to receptor site 5 of voltage-gated Na^+^ channels [2, 3]. Both parent compounds and their derivatives inhibit channel deactivation [4]. Because they affect voltage-gated Na^+^ channel activity, brevetoxins are responsible for neurotoxic shellfish poisoning (NSP), may cause marine animal mortalities during blooms, and have been implicated in fish kills [5]. Additionally, *K. brevis* cells that are damaged by the breaking surf have been shown to release enough aerosolized brevetoxins to cause eye irritation and respiratory distress in humans near the shore [6].

Despite the health risks associated with brevetoxins, their biological function within *K. brevis* is currently unknown. Notably, recent evidence suggests that PbTx-1 and PbTx-2 production increases in response to hypo-osmotic stress. Within 24 hours after a rapid media salinity reduction (35 to 27), the *K. brevis* Wilson clone (CCFWC268) produced ~25% more total brevetoxin per cell. It was therefore hypothesized that toxin production facilitates the response of *K. brevis* to salinity variations in the natural environment [7]. As populations move from offshore oceanic to coastal waters, cells experience a range of environmental salinities. For example, cells of this oceanic species have even been observed in the hyposaline Mississippi River Delta [8].

To better characterize brevetoxin production and osmoacclimation in *K. brevis*, the reference transcriptomes of three *K. brevis* clones, Wilson-CCFWC268, SP1, and SP3 were assembled. These clones represent diverse geographic origins, duration of years in culture, and toxin profiles. Wilson was initially collected off the coast of Florida in 1953. In contrast, the SP1 and SP3 clones originate from the Texas coast in 1999. While SP1 produces low, often undetectable amounts of PbTx-1 and PbTx-2, the total brevetoxin in SP3 and Wilson consistently exceeds 10 pg cell^-1^ [7, 9]. Thus, differences among the three transcriptomes may improve our understanding of brevetoxin production.

Brevetoxins are ladder-frame polyethers that belong to the polyketide family. Polyketide synthase (PKS) genes have been isolated from Wilson cultures [10]. In 2008, Monroe *et al.* identified four novel *K. brevis* PKS mRNA sequences in *K. brevis* clones Wilson, C2, NOAA-1, and SP2 that were not present in other dinoflagellates, including closely-related *Karenia mikimotoi* and *Karlodinium veneficum*. Because these four PKS sequences are unique to *K. brevis* and are structurally novel, it was hypothesized that they participate in the brevetoxin biosynthetic pathway [11]. As part of this project, we searched the SP1, SP3, and Wilson transcriptomes for novel *K. brevis* PKS genes to determine if the known PKS genes were transcribed in all three clones.

Because brevetoxins increase voltage-gated Na^+^ channel activity, determining whether *K. brevis* expresses these channel proteins may elucidate the physiological function of this toxin. Previously constructed *K. brevis* EST libraries contained ~12,000 unique genes [12, 13], but no complete Na^+^ channel protein-coding region was identified in this gene set. Further, channel-based Ca^2+^, K^+^, and Na^+^ transport across cell membranes is one known method that plants and algae use to maintain homeostasis [14]. The presence of ion channel sequences in *K. brevis* transcriptomes would suggest that this dinoflagellate is capable of osmoregulation through selective transmembrane ion transport.

Although aquaporins and VATPases have not been implicated in brevetoxin binding, they might affect osmoacclimation efficiency in *K. brevis*. Aquaporins were originally discovered in human red blood cells [15] but have since been found in taxa belonging to the bacterial, archaeal, and eukaryotic domains [16]. These bidirectional transport proteins belong to the major intrinsic protein (MIP) family and move water and/or glycerol molecules across lipid membranes more quickly than diffusion [17, 18]. Similarly, VATPases generate pH gradients that trigger secondary ion transport [19]. They have been identified in diverse eukaryotes and may participate in osmoregulation. For example, in *Arabidopsis thaliana*, Ca^2+^, Na^+^, and K^+^ starvation induced transcript-level downregulation of VATPase family genes [20], and inhibition of plasma H^+^‒ATPases in green alga *Dunaliella salina* prevented cell volume recovery after hyper-osmotic stress [21].

The haploid *K. brevis* genome is estimated to be 1 × 10^11^ base pairs (bp) [22–26] and has not been sequenced. This exceedingly large genome size highlights the crucial need for a reference transcriptome in order to initiate serious genomic analyses of this species. The Wilson, SP1, and SP3 *K. brevis* transcriptomes are among the first dinoflagellate transcriptomes to be assembled. Because no reference genome was available, a goal of this study was to evaluate different techniques to determine the *de novo* assembly method best suited for our data. Among eukaryotes, dinoflagellates are unique in a number of ways. A 22-nucleotide spliced leader sequence (SLS) has been identified in nuclear mRNA from all dinoflagellate species, including *K. brevis* [13, 27]. Though the dinoflagellate SLS is conserved within the dinoflagellate group, it is not homologous to SLSs used by other eukaryotic phyla [27]. Additionally, dinoflagellate chromosomes are permanently condensed and nuclear genomes often contain a high quantity of repetitive, non-coding DNA [23, 28, 29]. These characteristics make dinoflagellates a biologically interesting target for transcriptome characterization, analysis of the gene complement, and gene expression studies. Here we report the results of a search for *K. brevis* PKSs, voltage-gated ion channels, aquaporins, and VATPases and describe transcriptome sequence differences in these genes among ecologically diverse clones.

## Results

### Transcriptome assembly

**Figure 1:**
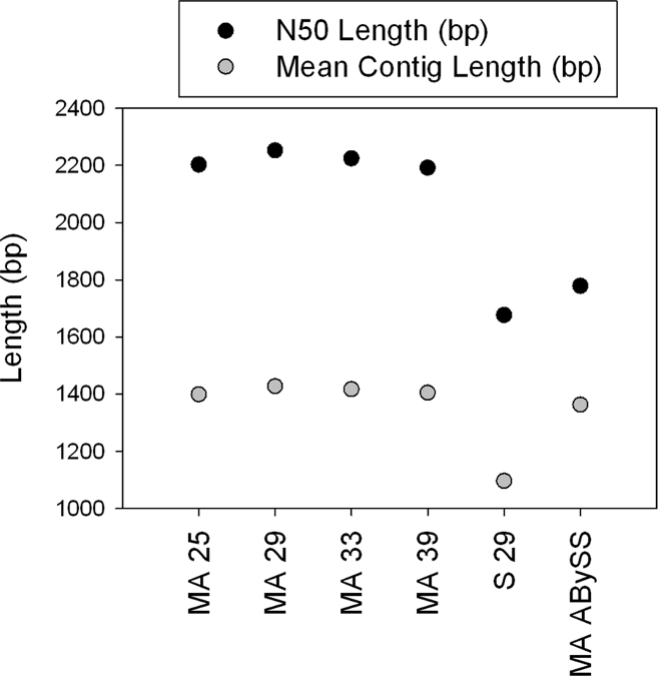
**N50 and mean transcript length values for merged (MA) and single k-mer (S) Velvet-Oases and ABySS SP1 transcriptome assemblies.**

After trimming for quality and length, 58.5 million, 78.0 million, and 51.4 million paired reads were used to assemble the *K. brevis* Wilson, SP1, and SP3 reference transcriptomes, respectively (Table 1) using both the Velvet-Oases [30, 31] and ABySS [32] assembly methods. Based on the N50 length and mean transcript length (Figure 1), the transcriptomes produced by the Oases merged assembly (MA) technique with K-values of 29 bp (Wilson and SP1) or 33 bp (SP3) were considered optimal and used during all subsequent analyses. Further, the transcript analysis software TRAPID [33] identified 1549 more full-length open reading frames (ORFs) in the SP1 MA Oases transcriptome, compared to the SP1 MA ABySS transcriptome. These results support the choice of Velvet-Oases, since this assembler produced more transcripts with complete protein-coding regions.

**Table 1:** **A comparison of** ***K. brevis*** **transcriptome read number, locus number, apparently clone-unique locus number, N50 length, and mean locus length values**

Clone	# Reads	# Loci	% Isoforms	# Unique loci	N50 (bp)	Mean locus length (bp)
Wilson	58,535,595	86580	34	3712	2038	1340
SP1	77,994,379	93668	35	8202	2124	1376
SP3	51,363,303	84309	85	2606	3424	1941

The % isoforms column lists the percentage of loci that were assigned two or more isoforms by Velvet-Oases.

**Figure 2:**
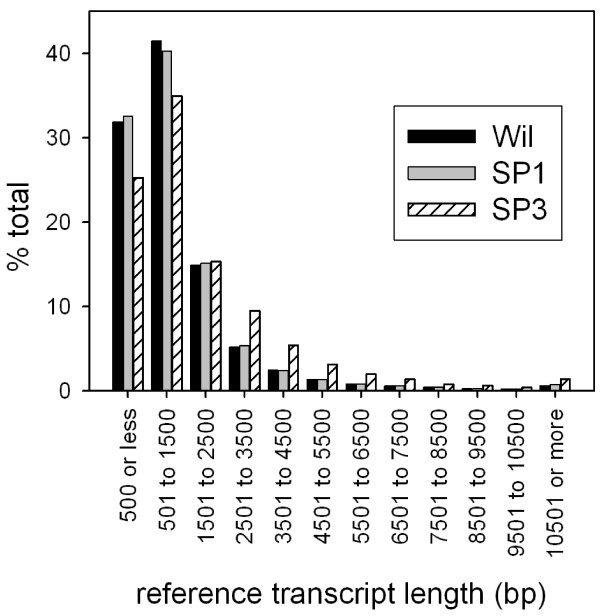
***K. brevis*** **transcriptome reference transcript length histogram.**

The reference transcriptomes had ~ 90,000 loci each (Table 1). Of these, 34% (Wilson), 35% (SP1), and 85% (SP3) contained two or more isoforms that were collapsed to a single representative transcript (Table 1). Approximately 87% (Wilson and SP1) or 74% (SP3) of the transcripts were less than 2,500 bp long (Figure 2). Based on BLASTn results, 4.3% (Wilson), 8.8% (SP1), and 3.1% (SP3) of the loci in each transcriptome were present in only one of the clones (Table 1). However, when reads were aligned to the assembled transcriptomes, < 1% of the transcripts only matched reads from one clone (Table 2). This suggests that the BLASTn results overestimate the number of loci apparently unique to Wilson, SP1, or SP3.

**Table 2:** **Short read alignment results**

Transcriptome	# Transcripts without Wilson alignments	# Transcripts without SP1 alignments	# Transcripts without SP3 alignments
Wilson	0	472 (0.55%)	438 (0.51%)
SP1	751 (0.80%)	0	718 (0.77%)
SP3	610 (0.72%)	564 (0.67%)	0

### Whole-transcriptome annotation

**Figure 3:**
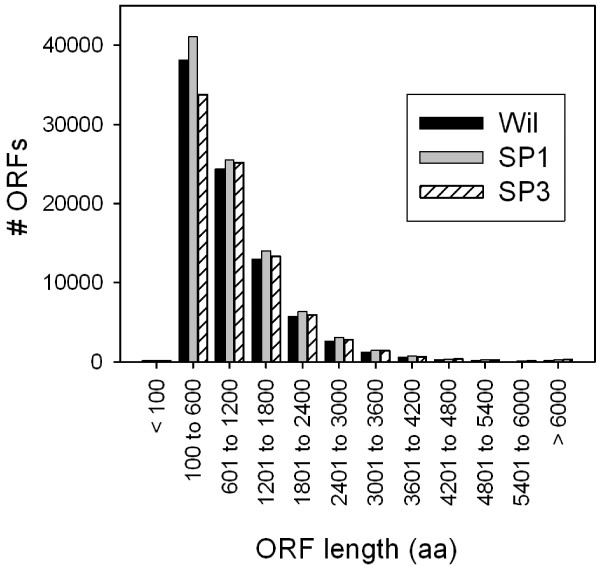
**Predicted ORF length distribution in the Wilson, SP1, and SP3 transcriptomes.** Length values are represented in # amino acids, or # bp in ORF divided by three.

**Figure 4:**
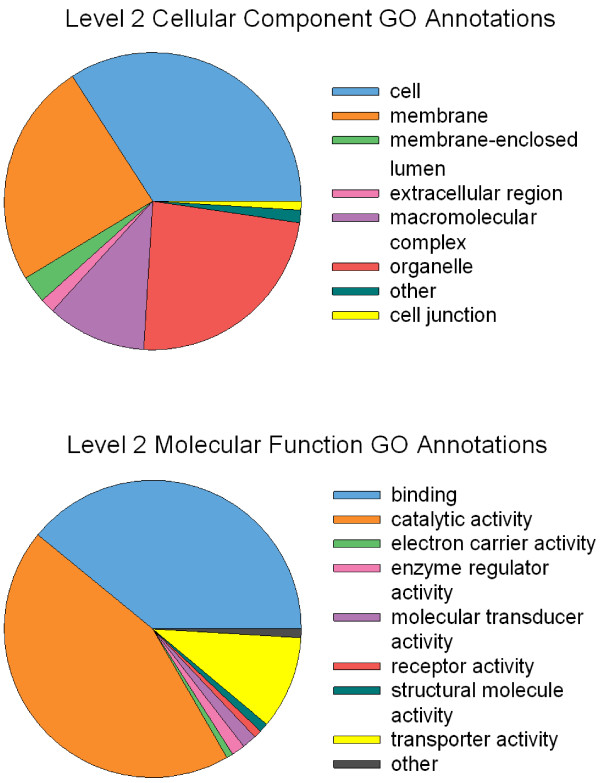
**Distribution of second-level cellular component and molecular function GO annotations in annotated** ***K. brevis*** **reference transcripts.** The percent distribution is identical in all three clones.

The number of predicted ORFs and their length distribution was similar among the three transcriptomes (Figure 3). Complete (start to stop codon) and partial (no start codon) ORFs longer than 300 bp were considered to be possible protein-encoding transcripts. During the BLASTp search against the nr database, 45% (Wilson), 43% (SP1), and 43% (SP3) of the *K. brevis* putative peptides > 100 aa significantly hit a sequence (E value ≤ 1.00E-6). Of those transcripts with positive BLAST alignments, 77% (Wilson), 77% (SP1), and 73% (SP3) were annotated with GO terms (Figure 4).

### CEGMA and TRAPID analyses

Core eukaryotic genes (CEGs) represent an unbiased set of proteins that are expressed and conserved within diverse eukaryotes [34], and therefore CEG identification helps gauge transcriptome assembly completeness. With CEGMA, we found 81% (Wilson), 84% (SP1), and 82% (SP3) (Additional file 1) of the complete highly conserved core genes described by Parra *et al*. [34]. In comparison, 74% and 90% of the complete CEGs were identified in the dinoflagellate *Karlodinium micrum CCMP2283* transcriptome and diatom *Thalassiosira pseudonana* genome, respectively. Thus, as a metric of transcriptome assembly success, the identification of complete CEGs in our transcriptomes indicates a high level of completeness in their coverage. The analysis also identified many conserved proteins that can be used to refine dinoflagellate phylogenetic relationships.

The “missing” CEGs that were not identified in any *K. brevis* reference transcriptome may represent genes that are not used by dinoflagellates. It is also possible that “missing” *K. brevis* CEG orthologs are not highly conserved by CEGMA criteria. To investigate these options, the SP1 transcriptome was used to search the CEG protein list using BLASTx. SP1 transcripts hit 235 of the 248 core CEGs with an E value < 1.00E-6. Therefore, homologs of 25 of the 38 “missing” genes were detected in the CEGMA dataset using BLASTx.

The ratio of full-length/quasi full-length to partial coding regions predicted by TRAPID [33] characterizes protein coding region completeness. The Wilson, SP1, and SP3 Oases reference transcriptomes were compared against three databases: the OrthoMCLDB 5.0 alveolate clade db, the PLAZA 2.5 green plants clade db, and the OrthoMCLDB 5.0 *T. pseudonana* CCMP1335 species db. The ratios of complete to partial coding regions identified in the reference transcriptomes were 7.3:1 (Wilson), 13.5:1 (SP1), and 12.5:1 (SP3). Over 90% of the *K. brevis* transcripts that significantly matched sequences in the TRAPID databases had ORFs that fell within two standard deviations of the mean ORF length in their gene families.

### PKS, aquaporin, voltage-gated ion channel, and VATPase identification

The Wilson, SP1, and SP3 transcriptomes each included positive hits to the four novel *K. brevis* PKS sequences (gi # 148536478, 148536480,148536474, and 148536472), with nucleotide similarity values >99% over the aligned regions. No unique non-synonymous SNPs were identified in the SP1 PKS ORFs.

The same seven putative voltage-gated Na^+^ or Ca^2+^ channel genes were identified in all three *K. brevis* transcriptomes (SP1 transcriptome locus # 394, 12559, 19932, 26784, 30595, 36263, and 64946). Each sequence contained the voltage-sensing motif and four Pfam00520 domains with ~ six predicted transmembrane regions. During the BLASTx search against the nr database, the putative proteins very significantly (E value ≤ 1.00E-50) matched voltage-gated Na^+^ and Ca^+2^ channels from a range of organisms, including mammals.

The transcriptomes contained twelve different putative VATPase subunits with V-ATPase conserved domains. Two MIP genes were also identified in all the transcriptomes. The 267 and 405 aa putative proteins (SP1 transcriptome locus # 102528 and 12778, respectively) each contained six predicted transmembrane regions and conserved domains belonging to the MIP family. The aquaporin Asn-Pro-Ala (NPA) water selectivity filter motif was also identified, with conservation of the Asn in each occurrence. The *K. brevis* MIP sequences were compared to the nr protein database. Each returned over 100 hits with E values ≤ 1.00E-20. Of these, ~70% were aquaporins or predicted MIPs. The complete ORF of MIP #12778 was located in Wilson, SP1, and SP3, with almost 100% aa sequence conservation among clones, with just one aa variation in SP3, a Val to Met substitution at aa position 270. The ORF of MIP # 102529 was incomplete in the Wilson and SP3 transcriptomes.

### SNP identification

When the Wilson and SP3 short reads were aligned to the SP1 transcriptome with a conservative 20-fold minimum coverage cutoff, 186,075 SNP locations were identified in 30,227 loci. Of these, 75,176 (40%) were exclusively in Wilson, 65,867 (35%) were exclusively in SP3, and 45,032 (25%) were in both Wilson and SP3. The SNP frequencies between Wilson and SP1, Wilson and SP3, and SP1 and SP3 were 0.0023, 0.0024, and 0.0022, respectively (Table 3). The 10-fold threshold analysis identified 312,723 potential SNP locations in 58,051 loci. Among these, 117,714 (38%) were exclusively in Wilson, 111,660 (36%) were exclusively in in SP3, and 83,349 (26%) were present in both Wilson and SP3. At this coverage threshold, the estimated Wilson and SP1, Wilson and SP3, and SP1 and SP3 SNP frequencies had increased to 0.0028, 0.0030, and 0.0028 (Table 3).

**Table 3:** **SNP detection results**

	Clones	# SNPs	Mean SNP Rate	# Transcripts with SNPs
	Wilson vs SP1	120208	0.0023 (1/442)	25123
20X	Wilson vs SP3	141044	0.0024 (1/421)	28427
	SP1 vs SP3	110899	0.0022 (1/465)	22794
	Wilson vs SP1	201063	0.0028 (1/358)	37486
10X	Wilson vs SP3	229374	0.0030 (1/339)	41937
	SP1 vs SP3	195009	0.0028 (1/364)	36144

The mean SNP rate was calculated based on the sum of nucleotides in transcripts with at least one SNP.

SNPs were analyzed in the seven putative voltage-gated Na^+^ or Ca^2+^ channels to examine variations among similar genes. The channel ORF length ranged from 4401 to 7908 bp, with a mean length of 6110 bp. Because the mean coverage of each sequence exceeded 10-fold, SNP rates were determined by the 10-fold threshold analysis. The mean SNP rate of all seven channels was 0.0032 (SP1 to Wilson), 0.0037 (SP3 to Wilson), or 0.0027 (SP1 to SP3) (Table 4). Notably, clone-to-clone SNP rate varied among voltage-gated cation channel sequences. Channel 19932 was ~100% identical in Wilson, SP1, and SP3, containing only one predicted SNP. In contrast, pairwise comparisons of channel 394 predicted a SNP ~ 1 out of every 160 nucleotides (Table 4). Non-synonymous SNPs that altered the amino acid sequence in the putative Na^+^ or Ca^2+^ channels were less common than synonymous SNPs, occurring at frequencies ranging from 0.0018 to 0.00033, with mean frequencies of 0.00095 (Wilson to SP1), 0.000867 (Wilson to SP3), and 0.000667 (SP1 to SP3) (Table 4). Variations in the non-synonymous SNP prevalence among putative cation channels suggest that the channels may be subject to different selective constraints. Furthermore, because Wilson, not SP1, is the most divergent clone overall, toxin production does not appear to affect cation channel gene selection.

**Table 4:** **SNPs in putative voltage-gated Na** ^**+**^ **or Ca** ^**2+**^ **channel sequences**

	Channel ID	# SNPs in transcript	Mean SNP rate	Length transcript	Length ORF	Non-synonymous/synonymous SNPs in ORF
Wilson vs SP1	394	45	0.0065 (1/153)	6893	6686	0.23
	12559	30	0.0046 (1/216)	6475	6213	0.39
	19932	1	0.0002 (1/6464)	6464	6165	1.00
	26784	25	0.0041 (1/241)	6034	5862	0.27
	30595	23	0.0032 (1/316)	7274	5532	—
	36263	20	0.0025 (1/401)	8014	7908	0.26
	64946	6	0.0013 (1/755)	4532	4401	0.80
Wilson vs SP3	394	44	0.0064 (1/157)	6893	6686	0.29
	12559	35	0.0054 (1/185)	6475	6213	0.25
	19932	0	0	6464	6165	—
	26784	27	0.0045 (1/223)	6034	5862	0.23
	30595	24	0.0033 (1/303)	7274	5532	0.00
	36263	19	0.0024 (1/422)	8014	7908	0.20
	64946	19	0.0042 (1/239)	4532	4401	0.19
SP1 vs SP3	394	39	0.0057 (1/177)	6893	6686	0.13
	12559	17	0.0026 (1/381)	6475	6213	0.29
	19932	1	0.0002 (1/6464)	6464	6165	1.00
	26784	22	0.0036 (1/274)	6034	5862	0.19
	30595	5	0.0007 (1/1455)	7274	5532	—
	36263	13	0.0016 (1/616)	8014	7908	0.18
	64946	21	0.0046 (1/216)	4532	4401	0.44

## Discussion

Our *K. brevis* transcriptomes are among the first dinoflagellate transcriptomes to be assembled, so it is not possible to make comparisons with closely related species. Lacking a reference genome sequence, several metrics, including transcript length, TRAPID-predicted full-length genes, and the identification of CEGs, were employed to gauge the completeness of the transcriptome assembly. Each of these criteria suggests that the transcriptomes are highly complete. First, the estimated mean protein-coding gene length of 19 model eukaryotes with sequenced genomes, including *Arabidopsis thaliana*, *Caenorhabditis elegans*, and red alga *Cyanidioschyzon merolae*, is 1,346 bp [35]. This value is similar to the Wilson, SP1, and SP3 mean reference transcript lengths of 1340, 1376, and 1941 bp, respectively; therefore, our *de novo* assemblies of the *K. brevis* transcriptome appear to yield transcripts of a reasonable length.

Next, a 66% CEG identification rate was reported when Parra *et al*. analyzed the *Toxoplasma gondii* genome with CEGMA [34]. Apicomplexan *T. gondii* and dinoflagellate *K. brevis* both belong to the alveolate group and are close phylogenetic relatives, based on rRNA analyses [36]. Identification of >80% of the highly conserved CEGs from each *K. brevis* reference transcriptome provides additional support for the completeness of the assembly, since this value exceeds the expected percent based on *T. gondii* results. TRAPID results also indicated that more transcripts contained complete or mostly complete ORFs than partial ORFs. Of the transcriptomes similar to TRAPID alveolate, gene families, over 92% were within two standard deviations of the expected length.

Only ~40% of the genes in the *K. brevis* transcriptomes are homologous to sequences in the nr protein database with a hit significance ≤ 1.00E-6. Because little is known about dinoflagellate genomes, the high percentage of unknown loci was expected. The Blast2GO annotation step did not annotate enough hits to allow conclusions about the total gene ontology distribution of the transcriptomes. The low annotation rate is a result of low (<50%) similarity scores between *K. brevis* sequences and annotated proteins. This may be the result of the phylogenetic uniqueness of dinoflagellates combined with limited phylogenetic representation in the Blast2GO databases.

The four novel *K. brevis* PKS sequences [11] were found and expressed in all three clones. No unique non-synonymous SNPs were identified in the SP1 PKS ORFs. It is therefore possible that SP1 expresses the genes involved in brevetoxin production, though cellular PbTx-1 and PbTx-2 are often undetectable in this clone. This result is consistent with prior work investigating transcriptional and post-transcriptional regulation in *K. brevis*. Microarray studies have observed a high percent of expressed genes related to RNA post-transcriptional processing and protein processing in *K. brevis*, thus suggesting that this dinoflagellate species is highly reliant on post-transcriptional regulation [26].

Some phenotypic variance between clones may result from SNPs that alter gene function. SNPs affecting 25123 (Wilson to SP1), 28427 (Wilson to SP3), or 22794 (SP1 to SP3) expressed genes were identified in laboratory-cultured *K. brevis* clones. Based on overall nucleotide divergence rates between Wilson and SP1, Wilson and SP3, and SP1 and SP3 (Table 3), SP1 and SP3 were the most similar clones. This may be the result of time in culture. SP1 and SP3 were isolated in 1999, while Wilson-CCFWC268 has been in culture since 1953 [9].

Typically, eukaryotic algae respond to osmotic stress by differential metabolite production rates and/or the transmembrane flux of ions and water [37]. The identification of putative MIPs, VATPases and voltage-gated Na^+^ or Ca^2+^ channels in *K. brevis* supports the hypothesis that cells osmoregulate with transmembrane channels. In *K. brevis*, aquaporins may facilitate quick responses to changes in the osmotic pressure gradient. Further, putative voltage-gated Na^+^ or Ca^2+^ channels with voltage-sensing motifs may facilitate cation transport across the cell membrane in response to ion gradients. The high interspecies similarity of these protein sequences indicates functional conservation among *K. brevis* clones that show varying brevetoxin profiles. Future experimental work will need to confirm that aquaporins, VATPases, or ion channels are involved with osmoacclimation in *K. brevis*. Potential experiments may measure cell volume post hypo-osmotic stress with and without specific protein blockers.

## Conclusions

Our discovery of putative ion channel, aquaporin, and VATPase sequences supports the hypothesis that *K. brevis* cells use transmembrane proteins during osmoregulation and osmoacclimation. In the future, clone-to-clone and treatment-to-treatment comparisons of the expression of these and other novel genes using RNA-seq [38] will elucidate *K. brevis* responses to osmotic stress. The transcriptomes assembled during this study also provide a foundational reference for future differential expression and protein discovery work. Thousands of *K. brevis* transcripts have been assigned GO functions (Additional files 2, 3, 4, 5, 6 and 7), and over 40,000 *K. brevis* loci containing unknown hypothetical protein-coding regions > 100 aa long (~11 kDa) were assembled. Even if just a fraction of these transcribed loci encode functional proteins, our datasets identify a vast number of novel genes and gene-products. Future analyses of these genes will yield insights into the unique biology of *K. brevis*.

## Methods

### Cell culturing and RNA sequencing

*K. brevis* clones Wilson, SP1, and SP3 were maintained in L1 medium [39] at salinity 35. The medium was prepared with filtered (0.2 μm pore) and autoclave-sterilized sea water from the Flower Garden Banks region, Gulf of Mexico. For each clone, triplicate 1-L sterile glass bottles were inoculated with cells from laboratory cultures and maintained on a 12:12 hour light:dark cycle at 25°C. Cell counts were performed by light microscopy every other day to monitor growth rates.

During the late exponential growth phase, 500 ml were concentrated by centrifugation (800 × g, 10 min) and RNA was immediately extracted from the pellets in 40 μL of nuclease-free water with the Qiagen RNEasy Mini Kit (Qiagen Inc., Valencia, CA), in accordance with the manufacturer’s protocol. After final elution of RNA in 40 μL of nuclease-free water, samples were stored at -80°C until library preparation. The RNA concentration and purity of each extraction was estimated by measuring the absorption spectra of 2 μL sample aliquots with a NanoDrop Spectrophotometer.

The remaining culture in each bottle was diluted from a salinity of 35 to a salinity of 27 with nutrient-enriched Milli-Q water to simulate hypo-osmotic stress. Stressed cultures were incubated for one hour before RNA was extracted, as described above. RNA was shipped on dry ice to the National Center for Genome Resources Sequencing Lab (Santa Fe NM, USA) for paired-end Illumina sequencing (Illumina, San Diego CA, USA). Libraries were prepared with the TruSeq RNA Sample Preparation Kit (Illumina) using 2 μg RNA. Paired-end 50 bp reads were sequenced with the Illumina Hi-Seq 2000 platform.

### Reference transcriptome assembly

Paired-end reads are available at the NCBI SRA repository. Reads were trimmed for quality and filtered for length with CLC Genomics Workbench 5.5.1 (CLC Bio, Aarhus, Denmark); the minimum read length permitted was 45 bp. Reads were trimmed based on Phred quality scores at the probability threshold of p = 0.05.

Reference transcriptomes for each clone were assembled with reads pooled from both control and salinity stress treatments. Pooling the reads allowed the assembly of genes that may only be expressed in one of the two treatments. Assembly was completed with Velvet-Oases, which uses a de Bruijn graph algorithm to build transcripts *de novo* [30, 31]. Coverage cutoff values were chosen automatically, and the edge fraction cutoff was increased from the default 10% to 50%. For each clone, single k-mer assemblies (k-mer lengths 21, 25, 29, 33, 37, and 41 bp) were merged into a non-redundant consensus transcriptome assembly. A 250 bp minimum transcript length threshold was enforced. For comparison purposes, an SP1 reference transcriptome was also assembled with the ABySS *de novo* paired-end assembler [32]. Single k-mer assemblies (odd lengths, 25 to 45 bp) were merged into a final transcriptome with “bubble popping” enabled.

Oases may output several transcript isoforms belonging to the same predicted locus. When multiple isoforms were present, we removed all but one representative sequence based on the following criteria. Isoforms were searched using BLAST [40] against the other *K. brevis* transcriptomes, with the E value significance threshold 1.00E-6. The isoform that hit another sequence with the longest alignment length and highest significant bit score was retained. In the event that a locus containing multiple isoforms did not return a significant hit, the longest transcript was chosen to represent its locus. This technique retains the isoform that is most abundant across all clones, when multiple isoforms were present.

The mean locus (unigene) length and N50 value were calculated using a transcript length list. To identify putative complete genes, the transcriptomes were analyzed with TRAPID. Transcripts were assigned gene families by a comparison with the TRAPID alveolate clade database. ORFs within two standard deviations of the mean gene family length were considered “full-length”. For each *K. brevis* clone, the transcriptome assembly method that produced the greatest N50 length and the most full-length genes was considered optimal and used during subsequent analyses.

### Identification of core eukaryotic proteins

To assess transcriptome completeness, loci were analyzed with the Core Eukaryotic Genes Mapping Approach (CEGMA) pipeline. CEGMA was developed to identify a subset of 248 highly conserved core eukaryotic genes (CEGs) in eukaryotic genomes. The CEGs were derived from six diverse model organisms: *Homo sapiens, Drosophila melanogaster, Arabidopsis thaliana, Caenorhabditis elegans, Saccharomyces cerevisiae* and *Schizosaccharomyces pombe* [34]*.* For comparison purposes, the *T. pseudonana* genome was downloaded from the Joint Genome Institute and analyzed with CEGMA. *T. pseudonana* is a eukaryotic oceanic alga, for which a complete genome is available. Additionally, the *K. micrum* CCMP2283 transcriptome was downloaded from the CAMERA Data Distribution Center and analyzed with CEGMA. *Karlodinium* dinoflagellates are close phylogenetic relatives of *K. brevis*, based on rRNA analyses [41].

### Assessing gene completeness with TRAPID

Full-length, quasi full-length, and partial protein coding regions were predicted in the Wilson, SP1, and SP3 Oases reference transcriptomes and the SP1 ABySS merged assembly. All the transcriptomes were compared against sequences in the OrthoMCLDB 5.0 alveolate clade database, while the Oases reference transcriptomes were also compared against sequences in the PLAZA 2.5 green plants clade database and the OrthoMCLDB 5.0 *T. pseudonana* CCMP1335 species database. The similarity search considered hits that yielded an E value < 1.00E-5 to be significant, and transcripts were annotated according to best hit values. Transcripts that hit one or more sequences in the TRAPID databases were qualified as “full-length”, “quasi full-length”, or “partial” based on the ORF length. ORFs that were more than two deviations shorter than the average ORF length of their assigned gene family (excluding the 10% longest and shortest sequences within the family) are “partial”. ORFs that are longer than the mean minus two standard deviations are “full length” if they also contain a start and stop codon and “quasi full-length” if they lack a stop and/or start codon [33].

### Predicting unique assemblies and SNP locations in the transcriptomes

To identify similarities and possible differences among clones, the transcriptomes were searched for transcripts that are present in just one or two out of the three clones. Unique assemblies may be caused by differences in transcript coverage or transcriptome assembly artifacts. First, each Velvet-Oases assembled *K. brevis* transcriptome was converted to a searchable BLAST nucleotide database. All transcriptomes were searched against each other with BLASTn. Only hits with E values ≤ 1.00E-50 were considered as significant matches. Next, Wilson, SP1, and SP3 paired-end reads were aligned to each transcriptome with CLC Genomics Workbench 6.5. During mapping, 85% similarity fraction and 90% length fraction cutoffs were enforced, as well as conservative mismatch (3), insertion (3), and deletion (3) costs.

Reads from Wilson and SP3 were mapped to the SP1 transcriptome and analyzed for SNPs with the CLC quality-based variant detection function. The following quality filtering criteria were enforced: neighborhood radius = 5, maximum gap and mismatch count = 2, minimum neighborhood quality = 15, and minimum central quality = 20. Variants also had to be present in both forward and reverse reads. Additionally, non-specific matches and broken pairs were ignored, and the program enforced a 20-fold minimum coverage threshold and 90% minimum variant frequency. The analysis was also run with less conservative but more inclusive 10-fold coverage threshold.

### Whole-transcriptome annotation and targeted gene discovery

The longest putative ORF was identified in each transcript and translated to amino acids (aa) via the longorf.pl Bioperl script [42]. Using BLASTp, peptides were compared to the NCBI non-redundant protein database, which was downloaded from the NCBI FTP site on September 20, 2013. Significant (E value ≤ 1.00E-6) hits were uploaded to Blast2GO and annotated with possible gene ontology (GO) terms using a 35% minimum similarity cutoff [43].

In order to identify novel *K. brevis* PKS mRNA sequences, BLASTn was used to search the Wilson, SP1, and SP3 transcriptomes for the four novel *K. brevis* mRNA sequences identified by Monroe and Van Dolah [11]. Hits were aligned against each other with the Clustal Omega alignment program [44] to investigate sequence similarity among the clones and original PKS sequences.

To identify possible voltage-gated cation channels, VATPase subunits, and aquaporin transcripts, the transcriptomes were compared to annotated voltage-gated Na^+^ channel alpha subunit and aquaporin sequences using BLASTx.

During the targeted search for these particular genes, loci with significant (E value ≤ 1.00E-6) matches to one or more proteins from the databases were translated into ORFs. Any locus containing an ORF < 300 bp long was discarded.

Probable transmembrane domains within the complete ion channel and aquaporin ORFs were identified with the Center for Biological Sequence Analysis TMHMM Server, version 2.0, which uses a hidden Markov model approach to predict transmembrane, intracellular, and extracellular regions in proteins [45]. Since cation channels and aquaporins each contain domains with six transmembrane regions, sequences containing at least six predicted transmembrane helices were analyzed with two additional tools. First, they were compared to the NCBI nr database with BLASTx to identify homologous sequences from a range of eukaryotes. Next, conserved protein domains from the NCBI Conserved Domain Database [46] were identified with CD-search [47]. In particular, ion channel transcripts were expected to contain domains belonging to the Pfam ion transport protein family (Pfam00520), which includes Na^+^, K^+^, and Ca^2+^ channels. Aquaporin transcripts were expected to contain MIP family conserved domains. VATPases were searched for V-ATPase domains, including Walker motifs, which are conserved among VATPase A subunits [48].

Potential ion channel transcripts were also searched for the voltage-gated Na^+^ channel S4 voltage-sensing motif. This motif, a repeated triad containing one positively charged and two hydrophobic aa, is highly conserved and helps regulate conformational changes that occur during channel activation and inactivation [49].

## Availability of supporting data

The short read data supporting the results of this article are available in the NCBI SRA repository as of March 2014 under BioProject PRJNA214017, accession IDs SRX363776, SRX363775, and SRX361898. Short read data is also available at the publically accessible camera repository (project IDs MMETSP0573, MMETSP0574, MMETSP0648, MMETSP0649, MMETSP0527 and MMETSP0528). The reference transcriptomes can be accessed through LabArchive (doi:10.6070/H44F1NPC, 10.6070/H40P0X0H, 10.6070/H4VX0DH6).

Additional supporting data are included as additional files within the article (Wilson_CC_GO.tab, Wilson_MF_GO.tab, SP1_CC_GO.tab, SP1_MF_GO.tab, SP3_CC_GO.tab, SP3_MF_GO.tab, Wilson_CEG.txt, SP1_CEG.txt, SP3_CEG.txt).

## Electronic supplementary material

Additional file 1: **CEGMA CEG prediction results.** This document contains the CEGMA output describing the CEG analysis in the Wilson, SP1, and SP3 transcriptomes. (TXT 3 KB)

Additional file 2: **SP1 transcriptome cellular component GO annotation.** This document contains a full list of the Blast2GO GO cellular component terms assigned to SP1 transcripts. Data are arranged into six tab-separated columns: LevelGO, Term (Acc), Term (Name), #Seq, Score, Parents (Acc), Parents (Name). The Term (Name) and Term (Acc) columns contain the cellular component name and gene ontology ID number, respectively. #Seq lists the number of transcripts that were assigned the cellular component term. LevelGO, Parents (Acc), and Parents (Name) are all related to the hierarchal arrangement of gene ontology terms, where parents on lower levels branch into more specific, higher-level child terms. LevelGO therefore describes the specificity of the cellular component, where higher values are more specific, and the name and ID numbers of all its parents are listed in the Parents (Name) and Parents (Acc) columns. (TXT 62 KB)

Additional file 3: **SP1 transcriptome molecular function GO annotation.** This document contains a full list of the Blast2GO GO molecular function terms assigned to SP1 transcripts. Data are arranged into six tab-separated columns: LevelGO, Term (Acc), Term (Name), #Seq, Score, Parents (Acc), Parents (Name). The Term (Name) and Term (Acc) columns contain the molecular function name and gene ontology ID number, respectively. #Seq lists the number of transcripts that were assigned the cellular component term. LevelGO, Parents (Acc), and Parents (Name) are all related to the hierarchal arrangement of gene ontology terms, where parents on lower levels branch into more specific, higher-level child terms. LevelGO therefore describes the specificity of the molecular function, where higher values are more specific, and the name and ID numbers of all its parents are listed in the Parents (Name) and Parents (Acc) columns. (TXT 161 KB)

Additional file 4: **Wilson transcriptome cellular component GO annotation.** This document contains a full list of the Blast2GO GO cellular component terms assigned to Wilson transcripts. Data is arranged as described in Additional file 1. (TXT 59 KB)

Additional file 5: **Wilson transcriptome molecular function GO annotation.** This document contains a full list of the Blast2GO GO cellular component terms assigned to Wilson transcripts. Data is arranged as described in Additional file 2. (TXT 151 KB)

Additional file 6: **SP3 transcriptome cellular component GO annotation.** This document contains a full list of the Blast2GO GO cellular component terms assigned to SP3 transcripts. Data is arranged as described in Additional file 1. (TXT 61 KB)

Additional file 7: **SP3 transcriptome molecular function GO annotation.** This document contains a full list of the Blast2GO GO molecular function terms assigned to SP3 transcripts. Data is arranged as described in Additional file 1. (TXT 154 KB)
